# Targeting of Inhaled Therapeutics to the Small Airways: Nanoleucine Carrier Formulations

**DOI:** 10.3390/pharmaceutics13111855

**Published:** 2021-11-03

**Authors:** Danforth P. Miller, Thomas E. Tarara, Jeffry G. Weers

**Affiliations:** Respira Therapeutics, Inc., 5901 Indian School Rd NE, #107, Albuquerque, NM 87110, USA; dmiller@cysteticmedicines.com (D.P.M.); ttarara@cysteticmedicines.com (T.E.T.)

**Keywords:** respirable agglomerates, inhaled corticosteroids, ciclesonide, particle engineering, dry powder inhaler, extrafine, total lung dose, Alberta idealized throat, idealized child throat

## Abstract

Current dry powder formulations for inhalation deposit a large fraction of their emitted dose in the upper respiratory tract where they contribute to off-target adverse effects and variability in lung delivery. The purpose of the current study is to design a new formulation concept that more effectively targets inhaled dry powders to the large and small airways. The formulations are based on adhesive mixtures of drug nanoparticles and nanoleucine carrier particles prepared by spray drying of a co-suspension of leucine and drug particles from a nonsolvent. The physicochemical and aerosol properties of the resulting formulations are presented. The formulations achieve 93% lung delivery in the Alberta Idealized Throat model that is independent of inspiratory flow rate and relative humidity. Largely eliminating URT deposition with a particle size larger than solution pMDIs is expected to improve delivery to the large and small airways, while minimizing alveolar deposition and particle exhalation.

## 1. Introduction

The respiratory tract is divided into two principal regions, the upper respiratory tract (URT) comprising the mouth, larynx, and pharynx, and the lower respiratory tract (LRT) comprising the trachea, bronchi, and lungs. For therapeutics administered via oral inhalation, the LRT is generally the target. Unwanted deposition of particles in the URT may lead to local adverse events (e.g., throat irritation, cough, dysphonia, and opportunistic infections). If orally bioavailable, drug deposited in the URT may also contribute to systemic adverse events.

Regional deposition within the LRT may also be critical for effective drug delivery [1]. Traditionally, the LRT has been divided into two principal regions: (1) the conducting airways comprising the large airways, bronchioles and terminal bronchioles which extend from generation 0 to generation 15, and (2) the respiratory zone comprising the respiratory bronchioles in generations 16–19 and the alveolar ducts and alveoli in generations 20–23. The conducting airways contain pseudostratified, ciliated airway epithelial cells that remove particles and pathogens by a process known as mucociliary clearance. As such, deposition in the conducting airways can be estimated using 24 h clearance studies with imaging techniques (e.g., gamma scintigraphy and single photon emission computed tomography). Insoluble particles that are not cleared on the mucociliary escalator within this time are assumed to be deposited in the respiratory zone (also referred to as peripheral deposition). Drug that is cleared over this time is deemed to be deposited in the conducting airways (also referred to as central deposition) [2].

During the past decade, there has been increasing evidence that the “small airways” (i.e., airways < 2 mm in internal diameter, comprising generations 8 to 19 in the respiratory tree) contribute substantially to the pathophysiology and clinical expression of asthma and chronic obstructive lung disease (COPD) [3,4,5,6,7]. The small airways span both the central and peripheral regions of the lungs.

For particles to be deposited in the lung periphery, they must first bypass inertial impaction in the URT and large airways, after which they must sediment in the small airways or alveoli before being exhaled [1]. The Stokes number (Stk) defines the tendency that a particle will diverge from the airflow and deposit by inertial impaction in the respiratory tract, Equation (1):

(1) $$Stk=\frac{\rho_{p}d_{p}^{2}u}{18\mu D}\sim \;\frac{d_{a}^{2}Q}{18\mu D}$$

where $d_{p}$, $\rho_{p}$, and $d_{a}$ are the particle diameter, density, and aerodynamic diameter, respectively, u and μ are the linear velocity and dynamic viscosity of the carrier gas, and D is a characteristic length scale equal to the diameter of the airspace. The volumetric flow rate, Q, is often used to approximate the linear velocity. The product $d_{a}^{2}Q$ is termed the “impaction parameter”.

The terminal settling velocity for particles in the lung periphery, υ, is also proportional to $d_{a}$, but is no longer dependent on Q, Equation (2):

(2) $$\nu =\frac{\rho_{p}d_{p}^{2}}{18\mu }g\;∼\;\frac{d_{a}^{2}}{18\mu }g$$

Here, g is the acceleration due to gravity.

Current marketed dry powder inhalers (DPIs) for the treatment of asthma and COPD comprise either adhesive mixtures of coarse lactose carrier particles (median diameter, X_50_ = 60–200 μm) and micronized drug particles (lactose blends, LB), or spheronized agglomerates (X_50_ ~ 100 μm) of micronized drug (SPH). These types of formulations exhibit bimodal particle size distributions, with the fine mode comprising free micronized drug particles, and the coarse mode comprising either agglomerated drug in SPH, or drug adhered to coarse carrier particles in LB. Unfortunately, dispersion of drug particles from the non-respirable agglomerates in these formulations is poor, with 50–90% of the emitted dose lost in the URT [8].

**Figure 1:**
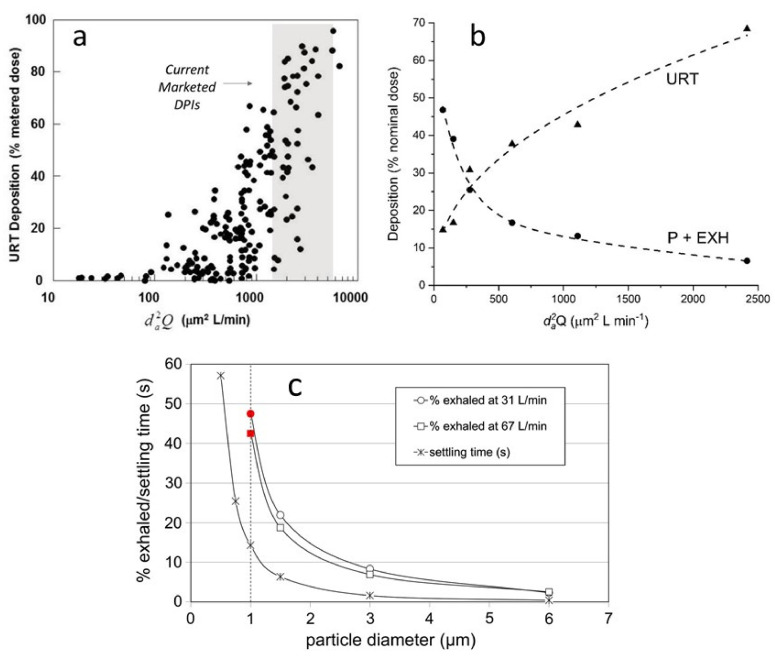
Particle deposition and exhalation in adult subjects as a function of variations in the impaction parameter, $d_{a}^{2}Q$ and $d_{a}$: (**a**) data for monodisperse liquid aerosols from Stahlhofen et al. [8,9]; (**b**) plot constructed from results of Usmani et al. for delivery of monodisperse particles of albuterol to adult subjects with mild asthma [28]; (**c**) comparison of the particle fraction exhaled and the time needed to sediment a distance 0.43 mm in a respiratory bronchiole at a stationary settling velocity. Both curves share the same ordinate. The figure is also constructed from results of Usmani et al.’s study [28] and extrapolated to 1 μm particles [29].

An empirical relationship between the impaction parameter and deposition in the URT of adults was first established by Stahlhofen et al. for monodisperse aerosols [9]. The experimental data are plotted in Figure 1. The empirical fit to the data is given by, Equation (3):

(3) $$URT\;Deposition=[1-(4.17\times 10^{-6}\left(d_{a}^{2}Q{)}^{1.7}+1{)}^{-1}\right]\times 100$$

As expected, increases in $d_{a}^{2}Q$ lead to corresponding increases in URT deposition. The shaded area in Figure 1a represents the range of $d_{a}^{2}Q\;$values of current marketed products comprising SPH and LB formulations ($d_{a}^{2}Q$ ~ 1452 to 5286 μm^2^ L min^−1^) [8]. This range of $d_{a}^{2}Q\;$values was calculated from the 50–90% mean URT deposition observed with these products and Equation (3).

For $d_{a}^{2}Q$ values between about 1500 to 3000 μm^2^ L min^−1^ (i.e., the sweet spot for lactose blends and spheronized particles), URT deposition for individuals may vary anywhere between 5% and 90% depending on the anatomical features of their mouth and throat [9,10]. Along the sigmoidal deposition curve, the variability in URT deposition decreases as $d_{a}^{2}Q$ increases above ~10,000 μm^2^ L min^−1^ (i.e., in the limit where all particles deposit in the URT), or as $d_{a}^{2}Q$ decreases to ~100 μm^2^ L min^−1^ (i.e., in the limit where most particles bypass deposition in the URT) [9].

The high variability in lung delivery also shifts the clinical dose response curve to higher nominal doses. While this helps to ensure that most patients achieve a therapeutic dose, it also results in additional drug being available to contribute to off-target effects.

In vitro anatomical throat models have recently been developed that enable estimates of URT deposition and the total lung dose (TLD) in various ages of subjects (e.g., adults, pediatrics, and infants) [11,12,13,14,15,16,17,18]. These models have demonstrated good in vitro-in vivo correlations for URT deposition and TLD for various inhaled drug products, making them a valuable tool in formulation design [19,20,21,22,23,24,25,26,27].

The influence of the impaction parameter on regional deposition within the lungs is less clear. Regional deposition of monodisperse liquid droplets containing albuterol was assessed for mild asthmatics with gamma scintigraphy by Usmani et al. [28] (Figure 1b). Consistent with Figure 1a, URT deposition increased with increasing $d_{a}^{2}Q$. Significant increases in peripheral lung delivery, as indicated by the sum of peripheral lung deposition and the fraction of drug exhaled (P+EXH) was observed as $d_{a}^{2}Q$ approached 100 μm^2^ L min^−1^.

Figure 1c shows the impact of decreasing $d_{a}$ on the probability of particle exhalation according to Equation (2) [29]. Note that subjects in this study did not perform a breath-hold following inhalation. For an extrafine particle with $d_{a}$ ~1.0 μm, the time for sedimentation within a 0.43 mm respiratory bronchiole is ~15 seconds. The sedimentation time decreases to less than 5 seconds for a particle with $d_{a}$ ~2.0 μm. Hence, the probability of achieving effective dose delivery to the peripheral regions of the lungs without particle exhalation increases for particles on the order of 2.0 μm. It also remains unclear what fraction of the peripheral dose is deposited in the respiratory bronchioles versus the alveoli. It is likely that the alveolar fraction increases with extrafine particles on the order of 1.0 μm compared to particles with a $d_{a}$ ~2.0 μm.

In previous studies we demonstrated how carrier-free formulations of spray-dried particles can effectively bypass URT deposition with just 2% to 5% extrathoracic deposition [8,30]. In the current manuscript we present results for a carrier-based formulation with a target $d_{a}$ and $d_{a}^{2}Q$ of ~2.0 μm and ~100 μm^2^ L min^-1^, respectively. As discussed, particles with these characteristics should largely bypass URT deposition with significant delivery to the small airways in the lungs. Although the utility of the technology is demonstrated in the context of the inhaled corticosteroid, ciclesonide, it can be applied to most therapeutics provided they are potent with a total lung dose less than ~10 mg.

## 2. Materials and Methods

### 2.1. Materials

Ciclesonide (batch CS-17007(JM-01)-001) was obtained from Aarti Industries Ltd. (Maharashtra, India). Water (HiPerSolv Chromano grade), Tween 20, and leucine were obtained from VWR Life Sciences (Solon, Ohio, USA). Glycerol and 2-propanol were obtained from Alfa Aesar (Ward Hill, MA, USA), while trifluoroacetic acid, methanol, and ethanol were obtained from JT Baker (Radnor, PA, USA). USP Perflubron (perfluorooctyl bromide, PFOB) was obtained from Atofina (Paris, France).

#### 2.1.1. Manufacture of Ciclesonide Powder for Inhalation (CPI)

The strategy around the design of an adhesive mixture with $d_{a}^{2}Q$ ~ 100 μm^2^ L min^−1^ is simple: eliminate the coarse fraction in the bimodal particle size distribution of conventional lactose blends. This may be achieved by developing a binary adhesive mixture of carrier and drug where drug-carrier and carrier-carrier agglomerates are respirable ($d_{a}$ < 5 μm). In such a circumstance there is no need to disperse the drug from the carrier to achieve effective aerosol delivery to the lungs.

The use of fine cohesive powders as carriers may seem counterintuitive given that lactose blends were developed to overcome the poor powder flow characteristics observed with fine, jet-milled drug particles sans carrier. However, the introduction of asperities in corrugated particles prepared by spray drying reduces interparticle cohesive forces sufficiently to enable: (1) accurate and precise filling of fine particles with drum-based filling machines, and (2) effective fluidization, emptying, and dispersion of powder agglomerates from passive DPIs [31,32,33].

**Figure 2:**
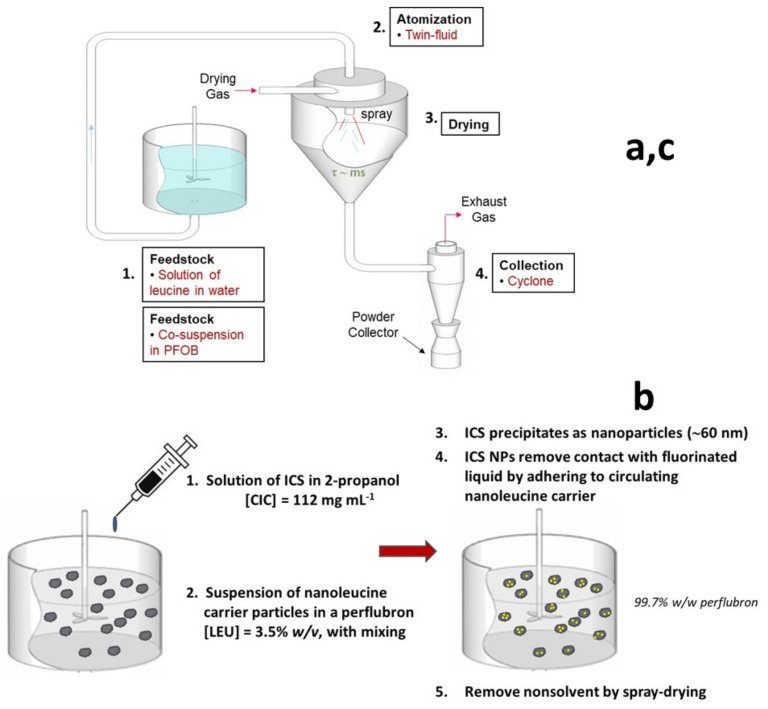
Process for the manufacture of adhesive mixtures of nanoleucine carrier particles and extrafine ciclesonide particles. (**a**) spray drying of leucine from a solution in water; (**b**) precipitation of nanoparticles of ciclesonide into a circulating dispersion of nanoleucine carrier particles in perflubron to form a co-suspension; (**c**) removal of perflubron to form a carrier-based dry powder comprising leucine nanoparticles adhered to nanoleucine carriers.

Adhesive mixtures of extrafine ciclesonide particles and leucine carrier particles (ciclesonide powder for inhalation, CPI) were manufactured via a three-step manufacturing process (Figure 2).

In step (a), fine leucine carrier particles were prepared by spray drying a 1.0% *w*/*v* solution of leucine in water. The solution was spray-dried on a Büchi B-191 spray dryer (Büchi, Flawil, Switzerland) with an inlet temperature of 110 °C, an outlet temperature of 65–70 °C, an aspirator setting of 100%, an atomizer gas pressure of 70 psi, and a liquid feed rate of 5.0 mL/min. A custom-built (Adams and Chittenden, Berkeley, CA) glass cyclone (1.75”) was used with a 1.25” diameter × 8” long collector.

In step (b), a concentrated solution of ciclesonide in isopropanol was prepared at about 50% of its solubility (112 mg/mL). Using an infusion pump (Harvard Apparatus, PHD2000) coupled with a precision 1.0 mL gas-tight syringe (Hamilton 81301), the ciclesonide solution was then added dropwise to a well-stirred large volume of perflubron (PFOB) containing a 3.5% *w*/*v* of a milky suspension of the leucine particles from step (a). The sudden change in solvent composition upon mixing of the alcohol and perflubron results in flash precipitation of extrafine ciclesonide particles. Although the size of the ciclesonide nanoparticles was not determined in the present study, previous studies exploring flash precipitation of inhaled corticosteroids in perflubron found a mean particle diameter of about 60 nm [34]. To achieve a target composition of 1% ciclesonide/99% leucine, only 0.15 mL of ciclesonide solution needed to be added to approximately 44 mL of perflubron. Thus, the combined solvent (perflubron and isopropanol) was predominantly perflubron (≈ 99.7% *v*/*v*). The very low solubility of ciclesonide in perflubron limits the potential for coarsening by molecular diffusion (Ostwald ripening) [35,36].

The leucine suspension in perflubron was mixed with a stir bar during alcohol addition. This process enabled uniform mixing of the drug and carrier particles, an outcome difficult or nearly impossible to achieve when mixing dry powders of this size with conventional low-shear and high-shear mixers [37,38]. Given their small size and desire to minimize interfacial contact with the fluorinated liquid, it is likely that the extrafine ciclesonide particles are rapidly adsorbed onto the carrier particles to form a co-suspension. The rapid association of drug and carrier to form a co-suspension in a fluorinated liquid was observed previously for micronized drug and PulmoSphere placebo particles in hydrofluoroalkane propellants [39].

In step (c), the nonsolvent is removed to form a dry powder. For this step, the Büchi B-191 spray dryer was utilized with the same hardware configuration as detailed above. The spray-drying conditions included an inlet temperature of 110 °C, an outlet temperature of 75 to 80 °C, an aspirator setting of 100%, an atomizer gas pressure of 70 psi, and a liquid feed rate of 5.0 mL/min. The resulting dry powder comprises an adhesive mixture of extrafine ciclesonide particles adhered to the leucine carrier particles.

#### 2.1.2. Capsule Filling of CPI

Size 3 hydroxypropylmethylcellulose (HPMC) clear capsules (Quali-V) were supplied by Qualicaps (Indianapolis, IN). The capsules were hand-filled with CPI to achieve a fill mass of ~6 mg. For the target 1% *w*/*w* ciclesonide content, this corresponds to a nominal dose of ~60 μg. This dose provides comparable deposition on stage 3 to filter in a Next Generation Impactor to that achieved with the 80 μg dose of the marketed Alvesco^®^ drug product.

### 2.2. Methods

#### 2.2.1. Primary Particle Size Distribution

Primary particle size distributions of the fine leucine carrier particles (< 5 μm) and their adhesive mixtures with extrafine ciclesonide particles (< 2 μm) were determined via laser diffraction (Sympatec GmbH, Clausthal-Zellerfeld, Germany) at a high dispersing pressure. The Sympatec H3296 unit was equipped with an R2 lens, an Aspiros micro dosing unit, and a RODOS/M dry powder-dispersing unit. Approximately 2–5 mg powder was filled into tubes, sealed and fed at a rate of 5 mm/s into the RODOS operated with 4 bar dispersion pressure and 65 mbar vacuum. Powders were introduced at an optical concentration of approximately 1% to 5% and data were collected over a measurement duration of up to 15 seconds. Particle size distributions were calculated by the instrument software using a Fraünhofer model. The particle size distributions are reported at three cut points in the distribution (i.e., X_10_, X_50_, and X_90_), which represent the volume-weighted size cutoffs for 10%, 50%, and 90% of the particles in the distribution.

#### 2.2.2. Tapped Density

Tapped densities were determined using a cylindrical cavity of known volume (0.593 cm^3^). Powder was filled into this sample holder using a microspatula. The sample cell was then gently tapped on a countertop. As the sample volume decreased, more powder was added to the cell. The tapping and addition of powder steps were repeated until the cavity was filled, and the powder bed no longer consolidated with further tapping. The tapped density is defined as the mass of the tapped bed of powder divided by the volume of the cavity.

#### 2.2.3. X-ray Powder Diffraction (XRPD)

X-ray powder diffraction patterns of selected powders were measured with a Rigaku Miniflex Model 600 diffraction system equipped with a D/Tex solid state “strip” detector (Rigaku, Tokyo, Japan). Each sample was prepared by packing bulk powder into a sample holder with a zero-background silicon insert. Samples were scanned from 3 to 35° 2θ at a scan rate of 1° 2θ/min, using a Cu radiation source with a wavelength of 1.54 Å, operated at 40 kV and 15 mA. During measurements, samples were rotated at 80 rpm.

#### 2.2.4. Dynamic Vapor Sorption (DVS)

The moisture sorption isotherm of CPI was measured at 25 °C using a dynamic vapor sorption (DVS) instrument made by Surface Measurement Systems, UK. This instrument gravimetrically measures uptake and loss of water vapor by a material. The DVS system is equipped with a recording microbalance with a resolution of ± 0.1 μg and a daily drift of approximately ± 1 μg. In the first step of the experimental run, the sample was dried at 25 °C and 0%RH for at least 600 minutes to bring the sample to a constant mass. Then, the instrument was programmed from 0 to 2%RH, to 5% RH, and then RH was increased in steps of 5% RH to 90% RH and decreased in steps of 5%RH from 90% to 0% RH. An equilibration criterion of dm/dt = 0.005%/min was chosen for the system to achieve at each RH step before automatically proceeding to the next RH step. Sample masses between 10 and 15 mg were used in this study.

#### 2.2.5. Ciclesonide Determination by RP-HPLC with UV Detection

Quantitation of Ciclesonide was done by reversed phase high performance liquid chromatography (RP-HPLC) with UV detection. An Agilent 1260 Infinity Series module HPLC system equipped with a UV detector (Agilent Technologies, Santa Clara, CA, USA) was used. Separation was achieved with an Agilent InfinityLab Poroshell 120 EC-C18, 3.0 × 150 mm, 2.7 μm column (P/N 693975-302) maintained at 40°C and gradient separation using water:trifluoroacetic acid (0.025% *v*/*v*) and acetonitrile:trifluoroacetic acid (0.025% *v*/*v*) operated at 0.6 mL/min. The autosampler was maintained at 2–8 °C and a 40 μL injection volume was used. Ciclesonide detection was performed at a wavelength of 242 ± 2 nm. Quantitation was performed by comparison to an external standard. Method linearity was established across a quantitation range from 0.08 to 200 μg/mL.

#### 2.2.6. Assay and Blend Uniformity

Assay testing was performed by weighing approximately 20 mg of formulated bulk powder onto a tared weighing paper. The weighed material was recorded and analytically transferred into a 25 mL volumetric flask following USP <1251> Method 3. The sample diluent (water/acetonitrile, 50/50 *v*/*v*) was used to rinse the residual materials into the flask. To evaluate assay/blend uniformity, three independent samples were weighed as described above. The samples represented different spatial locations from the container. The target nominal assay value was 10 μg ciclesonide/mg powder.

#### 2.2.7. Emitted Dose

The emitted dose (ED) represents the percentage of the nominal ciclesonide dose in the capsule that is discharged from the dry powder inhaler following device actuation. ED measurements were determined in accordance with USP <601> using a model 8601A Dose Unit Sampling Apparatus, DUSA (Copley Scientific Limited, Nottingham, UK). For ED measurements, a filled capsule (~6 mg fill mass; target 60 μg ciclesonide) was loaded into the AOS DPI inhaler and the capsule was pierced. The AOS DPI was then inserted into an aerosol mouthpiece adapter. A Copley model TPK2001 critical flow controller, and Copley model HCP vacuum pump drew air at a flow rate of ~27.7 L min^−1^ (2 kPa pressure drop) for a total volume of 2 L through the inhaler. Powder was emptied from the device into the DUSA equipped with a 47 mm A/E type glass fiber 1 μm filter (Pall Corp., Port Washington, N.Y., USA). The DUSA tube and filter were then extracted using 25 mL of sample diluent (water/acetonitrile, 50/50 *v*/*v*). The extract was then filtered through a 25 mm, 0.2 μm PTFE syringe filter. The mass balance of the ED test was assessed by also quantitating the residual ciclesonide in the actuated capsule and that deposited in the AOS DPI. The capsules and device were extracted using 2 mL and 5 mL of sample diluent, respectively. Each sample was quantitated for ciclesonide by RP-HPLC as detailed above and reported in terms of the percentage of the total recovered dose.

#### 2.2.8. Aerodynamic Particle Size Distribution (APSD)

Aerodynamic particle size distributions (APSD) were determined with a Next Generation Impactor (NGI) equipped with a USP induction port (MSP Corp. Minneapolis, MN, USA). Tests were conducted in accordance with USP <601> Aerosols “Aerodynamic Size Distribution, Apparatus 6 for Dry Powder Inhalers” and Ph. Eur. 2.9.18 “Preparations for Inhalation; Aerodynamic Assessment of Fine Particles; Apparatus E”. To prevent re-entrainment/bounce of particles within the NGI, the impactor stages were coated with a solution comprising 50% *v*/*v* ethanol, 25% *v*/*v* glycerol, 22.5% *v*/*v* water, and 2.5% *v*/*v* Tween 20. For stage 1 in the NGI, 2 mL of coating solution was used, 1.5 mL on stages 3 to 5, 1 mL on stages 2, 6, and 7, and 0.5 mL on the MOC (micro-orifice collector). APSD testing was conducted with the AOS^®^ DPI [39] at a pressure drop of 4 kPa, and an inhaled volume of 4 L. The 4 kPa pressure drop corresponds to a flow rate of ~40 L min^−1^ for the high-resistance AOS DPI (R ~ 0.051 kPa^0.5^ L^−1^ min). Quantitation of ciclesonide on each stage was performed by RP-HPLC as described above. Powder deposited within the induction port (IP) was extracted using 10 mL of sample diluent, NGI stages 1, 2 and MOC were extracted with 5 mL, and stages 2 through 7 were extracted using 10 mL of sample diluent. The fine particle fraction (FPF_<5__μ__m_) is reported as the percentage of active ingredient in the emitted dose with an aerodynamic size less than 5 μm.

#### 2.2.9. Mass Median Impaction Parameter (MMIP) and Stage Groupings

The mass median impaction parameter (MMIP) utilizes the impaction parameter cutoffs for the stages in an NGI as opposed to their size cutoffs [40]. Given that deposition within the respiratory tract depends on both the size and inhaled flow rate, MMIP provides a better correlate to regional deposition within the respiratory tract than does MMAD [41,42].

Fine particle fractions based on stage groupings (i.e., impaction parameters) are also reported here. The stage groupings include the “large particle fraction” comprising deposition on the USP induction port and stages 1 and 2 (mean $d_{a}^{2}Q$ > 1176 μm^2^ L min^−1^). When using the induction port, deposition on these stages contributes to extrathoracic deposition in the URT. The “airways fraction” refers to deposition on stage 3 through stage 5 (1176 ≥ $d_{a}^{2}Q$ ≥ 56 μm^2^ L min^−1^), while the “very fine particle fraction” refers to deposition on stages 6 to MOC ($d_{a}^{2}Q$< 56 μm^2^ L min^−1^). The extrafine fraction is thought to be largely associated with alveolar deposition, while deposition on stages 4 and 5 may be associated with small airways delivery. As pointed out by Dolovich et al. [43], “cascade impactors are not lung simulators”, and deposition of particles is far more complex than what a simple impactor can discern. Nonetheless, we are looking for gross differences in the APSD between various types of formulations with the hope of providing high level guidance on how these changes may impact deposition in the respiratory tract for an average subject. Ultimately, these hypotheses must be tested in vivo in patients in clinical studies. By using stage groupings as opposed to size cutoffs, we believe we have eliminated one major source of error in using cascade impactors for estimating regional lung deposition.

#### 2.2.10. Total Lung Dose

When a patient inhales through a passive DPI, the inhalation airflow generates the aerodynamic forces required to fluidize and deagglomerate powders into aerosols. These processes are complex to model, being highly dependent on formulation and device design, and sensitive to inhalation maneuver [44]. As such, it is generally easier to predict URT deposition in vitro by performing experiments using anatomical throat models.

The Alberta Idealized Throat (AIT) model represents an idealized version of the URT of an average adult subject [11,12]. The AIT geometry contains simplified analogues of anatomical features that heavily influence the transport and deposition of aerosols in the URT. The Idealized Child Throat (ICT) mimics the average deposition during oral inhalation among children 6−14 years of age [13,14]. Based on CT scans it appears that the main geometrical features of child and adult airways are similar. Hence, the ICT was simply adapted from the AIT with a scale factor of 0.62 [13,14]. Good in vitro/in vivo correlations have been established for pharmaceutical aerosols with these idealized and other anatomical throat models [15,16,17,18,19,20,21,22,23,24,25,26,27].

**Figure 3:**
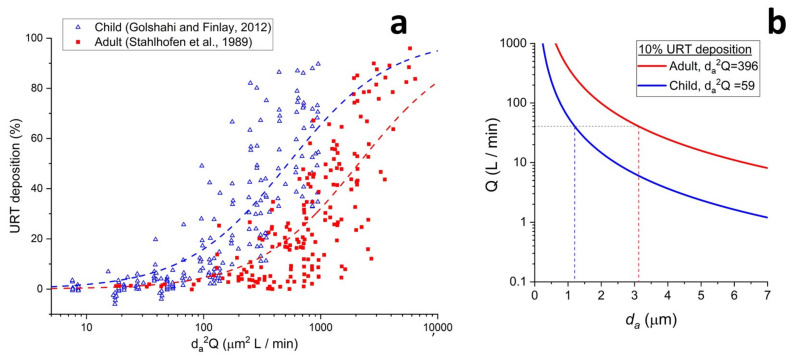
In vitro anatomical throat models. (**a**) Plot of URT deposition versus impaction parameter for child, and adult [9,13]. (**b**) Combination of flow rate and aerodynamic diameter that provide 10% URT deposition in adult and child throat models.

Figure 3a shows plots of deposition in children (ICT model) and adults (Stahlhofen data from Figure 1a) as a function of $d_{a}^{2}Q$. As the characteristic dimension of the airways decreases in younger subjects, equivalent URT deposition is shifted to smaller $d_{a}^{2}Q$ values. Hence, finer aerosols are needed to bypass URT deposition in children. The curve fit for URT deposition in the average child in the ICT model is presented in Equation (4) [13].

(4) $$URT\;deposition\;\left(ICT\right)=[1-1/\left(0.0001d_{a}^{2}Q+1)\right]\times 6$$

Figure 3b plots the data from Figure 3a in a different fashion, showing the combinations of Q and $d_{a}$ needed to achieve the target $d_{a}^{2}Q$ value required for 10% URT deposition. For a 39.2 L min^−1^ flow rate (4 kPa pressure drop with the AOS DPI), the required $d_{a}$ is about 1.2 μm in the ICT and 3.1 μm in the AIT.

Stainless steel versions of the AIT and ICT throats were obtained from MSP Corporation. For TLD measurements, the AOS DPI was coupled to the inlet of the throat using a custom mouthpiece adaptor, and the downstream end of the AIT was mounted to the filter housing stage of a Fast-Screening Impactor, FSI (MSP Corporation). The TLD that bypassed the throat was collected downstream on a 76 mm diameter A/E type glass fiber 1 μm filter (Pall Corp., Port Washington, NY). To prevent particle resuspension, the interior surfaces of the throats were coated with 15 mL of a solution comprising 50% *v*/*v* methanol and 50% *v*/*v* Tween 20. The coating solution was allowed to wet the internal walls of the AIT using a rocking or rotary motion to tilt the AIT from side to side. Excess coating solution was allowed to drain for 5 min before use.

During the TLD measurement, a filled CPI capsule was first loaded into the AOS DPI and pierced. A Copley model TPK2001 critical flow controller and Copley model HCP5 vacuum pump was activated to draw air at the desired pressure drop through the inhaler for a total volume of 2 L. The filter was removed from the FSI filter housing and placed in a liquid-tight plastic bag. The drug on the filter was then extracted using 20 mL of sample diluent (water/acetonitrile, 50/50 *v*/*v*). Each sample was quantitated for ciclesonide by RP-HPLC as detailed above and reported in terms of percentage of the total recovered dose relative to the average emitted dose. The mass balance of the test was assessed by quantitating the residual ciclesonide remaining in the actuated capsule and deposited in the AOS DPI. The capsules were extracted using 2 mL of sample diluent and 5 mL was used for the inhaler.

All ED, APSD, and TLD determinations were conducted under ambient laboratory conditions (~20–40% RH). Environmental robustness at high humidity was assessed by comparing the TLD measured with the ICT model at ambient conditions with TLD measured conducted in an environmental chamber (Barnstead International, Dubuque, IA, Model EC12560) at 25 °C/75% RH.

## 3. Results

### 3.1. Nanoleucine Carrier Particles

The leucine carrier particles utilized in the manufacture of the CPI batch described herein had a median geometric diameter ($X_{50}$) of 2.21 μm and a tapped density ($\rho_{tapped}$) of 0.038 g/cm^3^. It should be noted that these carrier particles are 25–100 times smaller than traditional lactose carrier particles. The geometric size and tapped density can be used to estimate the median aerodynamic diameter of the primary leucine carrier particles, $D_{a}$, using Equation (5) [8,30]:

(5) $$D_{a}=X_{50}\sqrt{\rho_{tapped}}$$

For this batch, $D_{a}$ is equal to 430 nm. Thus, from an aerodynamic perspective, the carrier particles can be described as “nanoleucine carrier particles”. Previous studies with spray-dried carrier-free formulations of proteins have demonstrated that for $D_{a}$ values between 300 nm and 700 nm, the agglomerates in the dry powder aerosol had an MMAD of about 1.8–2.0 μm, $d_{a}^{2}Q$ of about 105–135 μm^2^ L min^−1^, and URT deposition in the AIT model between 2–5% [8]. Hence, the primary nanoleucine carrier particles and their “respirable agglomerates” with other carrier particles are expected to be able to effectively bypass URT deposition.

### 3.2. Physicochemical Properties of Ciclesonide Powder for Inhalation

The physicochemical properties observed for a selected batch of CPI containing 1.0% *w*/*w* ciclesonide are detailed in Table 1. Additional batches with higher drug loadings (5, 10 and 20% *w*/*w*) were used for assessment of the physical form of the drug substance by XRPD.

### 3.3. CPI Particle Size Distribution

Given the low drug content (~1.0% *w*/*w*) and small size of the extrafine drug particles, the geometric size and tapped density of the adhesive mixture of drug and carrier is largely controlled by the size of the leucine carrier particles. Indeed, the $X_{50}$ (1.70 μm), $\rho_{\mathrm{tapped}}$ (0.047 g/cm^3^) and $D_{a}$ (370 nm) values in the formulated CPI drug product are all comparable to the values observed for the nanoleucine carrier particles detailed above. The low $D_{a}$ value suggests that both drug–carrier and carrier–carrier agglomerates are likely to be respirable. This is further supported by the low $X_{90}$ value of just 3.16 μm.

### 3.4. Drug Content and Blend Uniformity

One of the challenges with the development of dry powder blends of fine carrier particles and extrafine drug particles is the large increases in interparticle cohesive forces relative to gravitational forces that occur with decreases in particle size [33,45]. This results in poor powder flow properties as assessed by metrics such as the Hausner ratio or Carr’s index. This further leads to challenges in obtaining uniform blends with traditional powder mixing processes, such as low-shear and high-shear mixing. Whereas coefficients of variation in blend uniformity are less than 2% for conventional lactose blends, they can balloon to >30% for mixtures comprising fine carrier particles [37]. In contrast, the manufacturing process used for CPI achieves uniform mixing of the fine and extrafine particles by creating stable suspensions of the drug and carrier particles in a liquid that is a nonsolvent for the two materials. Mixing of these suspensions with low-shear mixers (e.g., overhead mixers) leads to excellent uniformity in drug content with an RSD of just 1.04 ± 0.01% *w*/*w* throughout the batch.

The unique blending process where the drug particles are created in a sea of carrier particles also limits the potential for development of drug-drug agglomerates, as is often observed with lactose blends. Free drug particles present in lactose blends may adhere to the walls of the receptacle (e.g., capsule or blister), or segregate from the carrier particles on storage [46]. This can be especially problematic for ultralow doses (~20 μg or less), where any losses in uniformity can adversely affect dose delivery.

The adhesive forces between drug and carrier in CPI are likely to exceed the dispersion forces achievable with DPIs. Hence, the drug is expected to remain adhered to the carrier during the inhalation process. The strong adhesive force between drug and carrier is comparable to or stronger than the adhesive forces observed in co-suspension formulations in pressurized metered dose inhalers [39]. There, the energy generated by rapid expansion of the propellant during actuation and passage through a small orifice in the actuator is insufficient to disperse micronized drug from the small porous PulmoSphere™ (re-branded Aerosphere^®^) carrier particles [39]. Given the small $D_{a}$ of the carrier particles with adsorbed drug, there is no need for the drug to be dispersed from the carrier to achieve effective lung delivery of CPI.

### 3.5. Crystallinity

**Figure 4:**
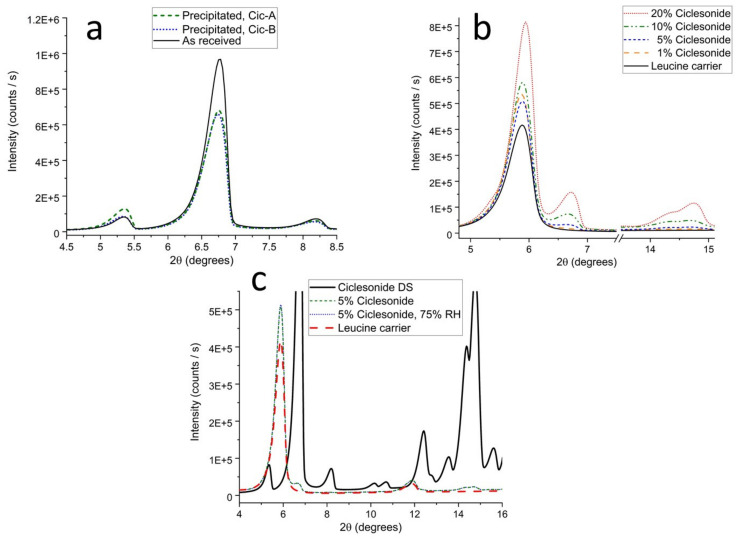
X-ray powder diffraction patterns for ciclesonide and leucine. (**a**) comparison of the diffraction patterns for the as received drug substance with two batches of particles prepared by flash nanoprecipitation (Cic-A, Cic-B); (**b**) comparison of the diffraction patterns at 1, 5, 10, and 20% *w*/*w* ciclesonide with the leucine carrier; (**c**) comparison of the diffraction patterns observed for a 5% *w*/*w* CPI drug product before and after exposure to 75% RH, with the patterns observed for the drug substance and leucine excipient.

X-ray powder diffraction (XRPD) experiments confirm that both the drug and carrier in the CPI drug product are highly crystalline (Figure 4). This is illustrated for ciclesonide by a comparison of the X-ray powder patterns of precipitated ciclesonide with that of the raw material (e.g., unprocessed starting material) (Figure 4a). The positions of the peaks indicate that the precipitated material is the same physical form (polymorph) as the as received ciclesonide drug substance (e.g., see the pronounced peak at 6.7°2θ). This crystalline polymorph of ciclesonide has been previously reported by Feth et al. [47]. These data also demonstrate the highly crystalline nature of the precipitated ciclesonide, as indicated by the lack of an amorphous background (“halo”).

For the 1% *w*/*w* blend, the concentration of ciclesonide is near the limit of detection for the (benchtop) X-ray diffractometer used. As expected, the diffracted intensity of the ciclesonide peaks increases with drug loading. Figure 4b shows an overlay of the X-ray powder diffraction patterns of CPI powders comprising 1, 5, 10, and 20% *w*/*w* ciclesonide. The X-ray powder diffraction patterns for different concentrations shows that the ciclesonide in the blends is crystalline, as indicated by the peak at 6.7°2θ, which could be detected for blends with a ciclesonide concentration ≥ 5% *w*/*w*. Upon enlargement of the powder pattern (not shown), weak diffraction peaks can be observed for the peaks at 14.9°2θ to 15.9°2θ of the 1% *w*/*w* ciclesonide powder. A qualitative assessment of the powder patterns in the blends indicates the highly crystalline nature of the blend formulation, as indicated by the lack of an amorphous background (“halo”). However, small amounts of amorphous material are difficult to detect via changes in the broad, diffuse background. A means to detect amorphous ciclesonide is to expose the sample to elevated relative humidity (RH) and then determine if increases in the intensity of diffraction peaks are present. The 5% ciclesonide/leucine blend was exposed to 75%RH for about 20 hours, an RH sufficiently high to depress the glass transition temperature (T_g_) of (any) amorphous ciclesonide and induce recrystallization. As shown in Figure 4c, the XRPD patterns before and after exposure did not change. This indicates that, within the limit of detection of the method, the ciclesonide/leucine blend contains no amorphous ciclesonide.

A sharp diffraction peak is also observed for leucine at about 5.7°2θ indicating the crystalline nature of the leucine carrier particles [48]. The crystallinity of the carrier particles is an important aspect of the design of such formulations. Highly crystalline materials tend to be non-hygroscopic, taking up very little water even at elevated relative humidity conditions. A highly crystalline formulation comprising hydrophobic materials is expected to provide improved environmental robustness in aerosol performance relative to formulations comprising amorphous or even hydrophobic phospholipid carrier particles. Indeed, the crystallinity of the CPI drug product is further illustrated by the low moisture sorption of the nanoleucine blend (< 0.2%) at 90% RH.

**Figure 5:**
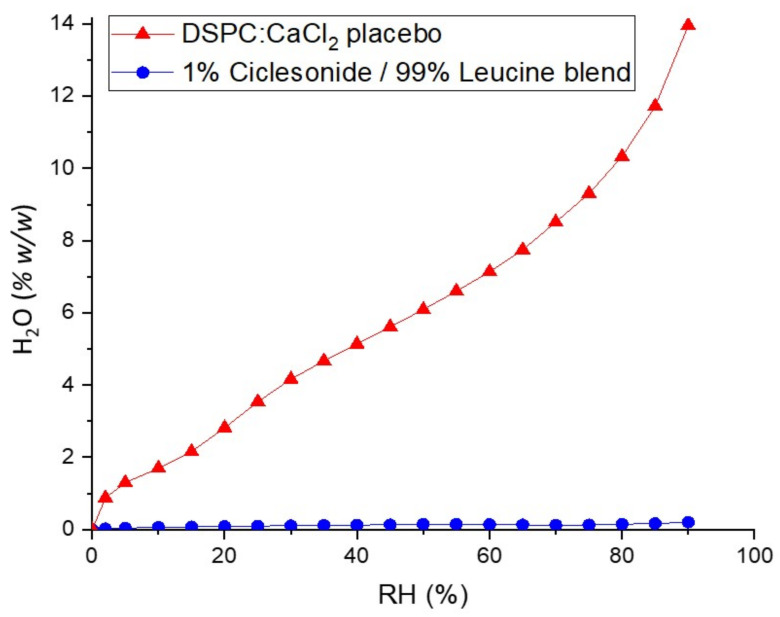
Moisture sorption isotherm (25 °C) of 1% *w*/*w* CPI in comparison with that of the standard PulmoSphere excipients comprising a 2:1 molar ratio of DSPC to calcium chloride.

Figure 5 compares the moisture sorption isotherms (25 °C) of CPI and spray-dried “benchmark” PulmoSphere carrier particles comprising a 2:1 molar ratio of distearoylphosphatidylcholine (DSPC) to CaCl_2_. Owing to the unique phase behavior of the amphiphilic long-chain phospholipid, the PulmoSphere placebo is considerably more hygroscopic. Indeed, at any RH, the DSPC:CaCl_2_ placebo is between 30 and 80 times more hygroscopic than the ciclesonide/leucine blend. Hence, the nanoleucine carriers are expected to provide more environmentally robust formulations than the PulmoSphere carriers studied previously [37,38,49,50]. Nanoleucine carrier particles may also provide improved environmental robustness for co-suspensions in pMDIs, provided they remain insoluble in the propellant.

### 3.6. Aerosol Performance of Ciclesonide Powder for Inhalation

Aerosol performance metrics for the CPI drug product are presented in Table 2.

The emitted dose with the high resistance AOS DPI at a pressure drop of 2 kPa (Q = 27.7 L min^−1^) was 94.0 ± 0.7%. Hence, despite the fine size of the low density, rugous carrier particles, the powder formulation fluidizes and empties from the capsule and device with high efficiency.

The APSD of CPI was assessed in an NGI at a pressure drop of 4 kPa (Q= 39.2 L min^−1^). The MMAD was 1.66 μm with a GSD of 1.57. The large particle fraction, comprising ciclesonide deposition within the induction port, stage 1, and stage 2, was only 2.3%, leading to a fine particle fraction less than 5 μm (FPF_<5__μ__m_) of 96.8%. The bulk of the deposition in the NGI was concentrated on stages 4 to 6, with limited deposition (4.4%) on stage 7 and MOC.

Given that each stage within the NGI represents a specific $d_{a}^{2}Q$ cutoff, it is instructive to characterize the particle distribution within the NGI based on stage groupings. The mass median impaction parameter (MMIP) for drug particles deposited within the NGI is 115.8 μm^2^ L min^-1^ (Table 2). It is important to note that the calculated MMAD and MMIP values comprise more than 98% of the emitted dose of drug. This contrasts with standard LB and SPH particles where only the fine mode of the bimodal distribution (comprising just 10–50% of the drug) is assessed in the impactor. Under this circumstance, the MMIP value for CPI is comparable to the $d_{a}^{2}Q$ values detailed in Figure 1 and Figure 3.

### 3.7. Total Lung Dose of CPI in Idealized Anatomical Throat Models

**Figure 6:**
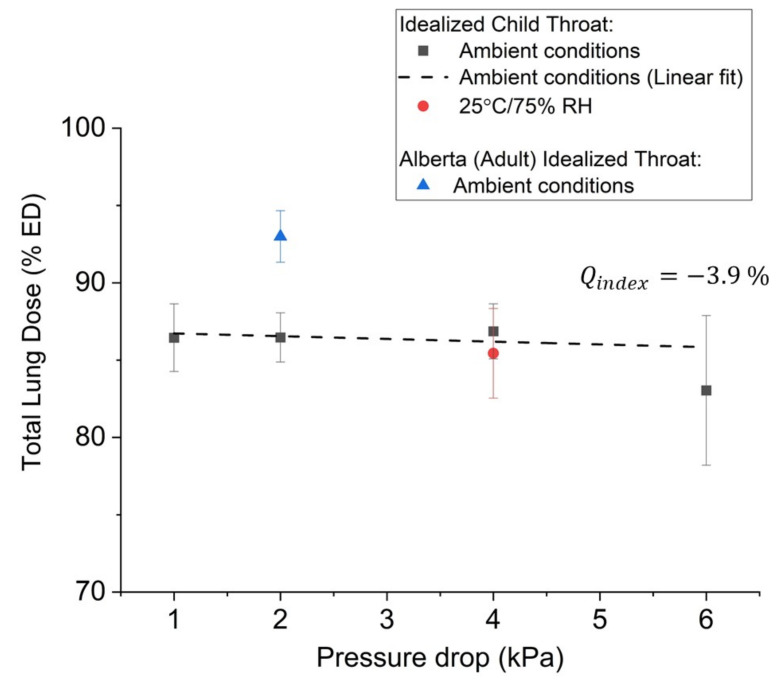
TLD measured in anatomical throat models as a function of pressure drop and variations in relative humidity.

The TLD was determined in both adult (Adult Idealized Throat, AIT) and pediatric (Idealized Child Throat, ICT) models, with mean values at a 2 kPa pressure drop of 93.0% and 86.5% of the ED, respectively (Table 2 and Figure 6). These results are consistent with the results observed in the NGI, and deposition predictions based on the measured MMIP/$d_{a}^{2}Q$ and Equations (3) and (4).

The dependence of the TLD with variations in pressure drop/flow rate was assessed in the ICT model at pressure drops of 1, 2, 4, and 6 kPa (Figure 6). This corresponds to flow rates ranging from 19.6 to 48.0 L min^−1^. Healthy seven-year-old children achieve a maximum inspiratory pressure (MIP) of about 5 kPa [51]. Subjects typically inhale at only 40–80% of their MIP values when using DPIs [51]. This corresponds to pressure drops between about 2 and 4 kPa in children. Indeed, most children ages six and above exceed a pressure drop of ~1 kPa when using passive DPIs [52,53,54,55]. This is especially true for children using higher resistance DPIs, as subjects tend to provide greater inspiratory effort when using a higher resistance device [56,57,58,59,60].

The flow rate dependence is expressed in terms of the *Q index*, a metric that assesses flow rate dependence in terms of the normalized difference in TLD at pressure drops between 1 kPa and 6 kPa [60]. The measured value of −3.9% is indicative of drug delivery that is largely independent of inspiratory flow rate.

Aerosol performance was also not impacted by increases in relative humidity, as the TLD measured at 75% RH in an environmental chamber was equivalent to that measured at ambient room temperature (20–40% RH). This is not surprising given the highly crystalline and hydrophobic nature of the drug and carrier, as illustrated by the very low oisture sorption characteristics detailed in Figure 5.

## 4. Discussion

Leucine is a hydrophobic amino acid that is being utilized as a shell-forming excipient in carrier-free spray-dried particles for inhalation [48,61,62,63]. The presence of leucine or its tripeptide trileucine on the particle surface improves powder dispersibility by lowering the particle density and the radius of curvature at interparticle contact points [48]. One benefit of leucine and trileucine is that the shell they form in engineered core-shell particles provides a protective barrier against the detrimental effects of elevated humidity, reducing the magnitude of the instantaneous drop in aerosol performance that is often observed for amorphous solids due to increased capillary forces between particles [64,65,66,67,68,69].

In this study, low density spray-dried microparticles of neat leucine are used as carrier particles in adhesive mixtures. These carrier particles are 25 to 100 times smaller geometrically, and owing to their low particle density, up to 500 times smaller aerodynamically than coarse lactose carrier particles. We use flash nanoprecipitation into a nonsolvent to load the nanoparticles of drug onto circulating carrier particles. The nonsolvent is then removed, leaving behind the carrier-based dry powder. The environmental robustness observed with carrier-free particles comprising leucine is also noted in these “nanoleucine” carrier formulations. The goal of achieving particles with a $d_{a}\;$of ~2.0 μm and a $d_{a}^{2}Q$ of ~100 μm^2^ L min^−1^ was met.

Despite their small size, these blends are easily fluidized and dispersed with a passive DPI. Based on in vitro aerosol performance metrics, the formulation is expected to largely bypass deposition in the URT and deliver high percentages of the nominal dose to the large and small airways, resulting in the safety/tolerability and dose consistency benefits detailed in the introduction.

### 4.1. Comparison of CPI to Marketed ICS Formulations

**Figure 7:**
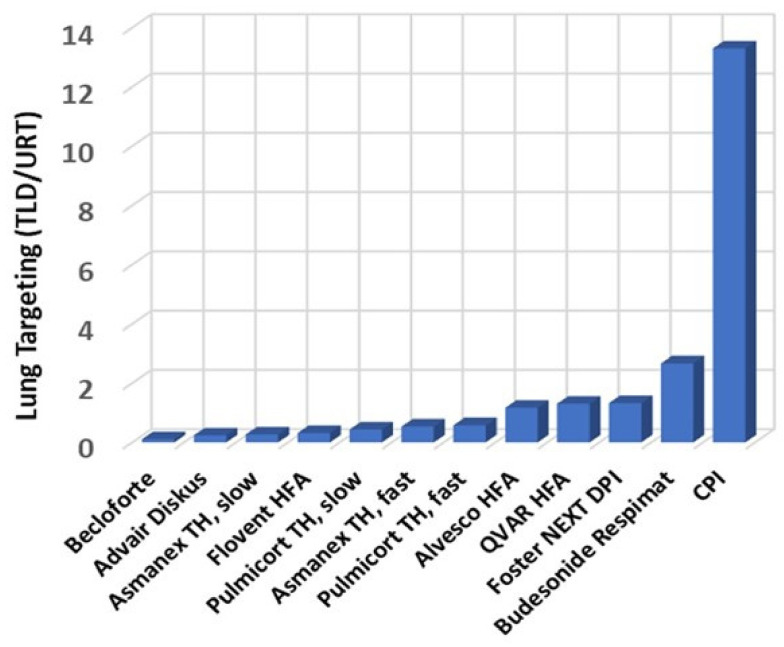
Lung targeting observed for CPI relative to various ICS formulations [60,70,71,72,73].

**Figure 8:**
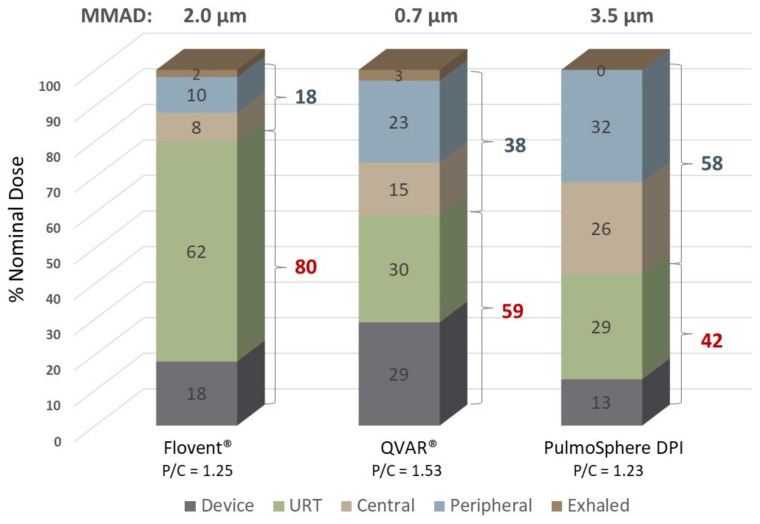
Comparison of regional deposition in the respiratory tract as determined by gamma scintigraphy for various ICS formulations [71,75].

**Figure 9:**
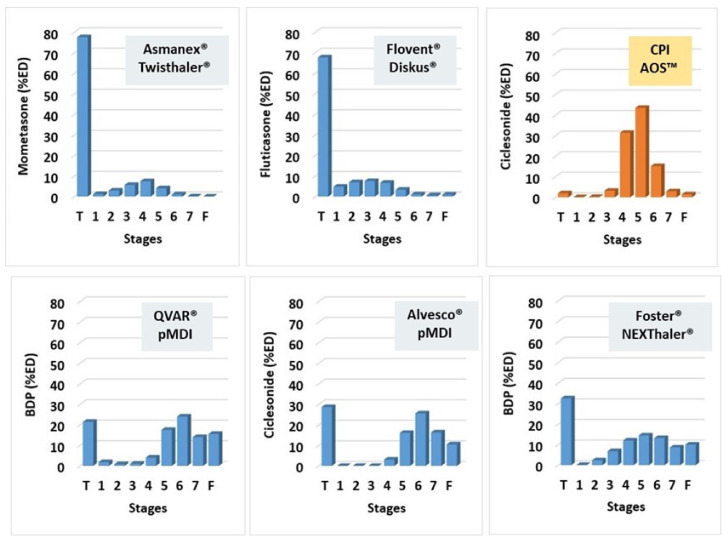
Aerodynamic particle size distributions of inhaled corticosteroid formulations [38,70,71,76].

To put the aerosol performance results observed with CPI in context, Figure 7, Figure 8, and Figure 9 and Table 3 compare these results with results obtained for marketed ICS products.

Figure 7 illustrates the improved lung targeting observed for CPI in adults relative to various marketed ICS formulations [60,70,71,72,73]. The plot leverages data from gamma scintigraphy and anatomical throat models. The results are expressed in terms of the ratio of LRT deposition (i.e., the TLD) to URT deposition. In the AIT model, the TLD/URT ratio for CPI is 13.3. This is more than five-fold greater than is achieved with the best marketed ICS product (i.e., budesonide administered with the high efficiency Respimat^®^ soft mist inhaler). Lung targeting with CPI is improved 55-fold relative to Advair^®^ Diskus^®^.

### 4.2. Regional Deposition in the Respiratory Tract

The transition from chlorofluorocarbon to hydrofluoroalkane propellants led to the development of solution-based pMDIs comprising extrafine ICS particles. Leach et al. demonstrated that 55–60% of the ex-actuator dose of an HFA solution formulation of BDP (QVAR, MMAD = 1.1 μm) was deposited in the lungs [74]. This was an astounding increase in TLD relative to the marketed CFC-BDP formulation at the time, where a 4–7% TLD was observed for fine particles with an MMAD of 3.5 μm. The extrafine QVAR formulation also deposited more than 30% of the ex-actuator dose in the peripheral lungs versus less than 3% for the CFC-BDP product [74].

Nonetheless it is important to point out that a formulation of extrafine particles is not a requirement for achieving high TLD and effective peripheral lung delivery of inhaled corticosteroids. This is illustrated in Figure 8 in a comparison of Flovent HFA (fine particle pMDI), QVAR (extrafine particle pMDI), and a carrier-free PulmoSphere dry powder formulation comprising fine spray-dried particles of budesonide [71,75]. Comparing the two pMDI formulations supports the conclusion that finer particles (MMAD of 0.7 versus 2.0 μm) leads to greater TLD (38% versus 18% of the nominal dose), increased peripheral lung delivery (23% versus 10%), and an increase in the ratio of peripheral to central lung delivery, P/C (1.53 versus 1.25).

The impact of aerodynamic diameter on regional deposition differs when a dry powder formulation that more effectively bypasses URT deposition is considered. Despite having an MMAD that is five times larger than that of QVAR, the TLD of the PulmoSphere DPI is increased from 38% to 58% and peripheral deposition is increased from 23% to 32% of the nominal dose. This is due to a significant decrease in the fraction of particles not sized in the impactor, i.e., those that are lost in the device and URT. Device and throat deposition is 59% of the nominal dose for QVAR versus 42% for the PulmoSphere DPI. For the extrafine CPI formulation in the present study, this fraction is markedly reduced to just 13% (AIT data).

While particle size is clearly important in achieving effective delivery to the lung periphery, developing formulations that reduce the large losses in the device and URT can be more impactful. Achieving improved lung delivery with larger sized particles may also reduce alveolar delivery and the probability of particle exhalation, as illustrated in Figure 1c.

The resistance of the device is another factor of critical importance in maximizing the TLD and peripheral lung delivery with DPIs. As discussed, inertial impaction is proportional to the impaction parameter, $d_{a}^{2}Q$. Subjects using higher resistance DPIs will inhale at lower flow rates, thereby decreasing inertial impaction. Given that inhaled flow rates with DPIs may vary from ~15 L min^−1^ to ~120 L min^−1^, the impact of device resistance on particle deposition can be significant. Additional work is needed, however, to establish the link between the impaction parameter, regional deposition within the lungs, and efficacy.

### 4.3. Small Airways Delivery

Figure 9 and Table 3 compare the pattern of stage deposition of CPI in an NGI with five marketed products comprising inhaled corticosteroids [38,70,71,72,76]. Each class of formulation (e.g., DPI formulations comprising fine particles, extrafine solution pMDIs and DPIs, and CPI) exhibit characteristic “fingerprints” in terms of their APSD. The top row of Figure 9 compares CPI with two market-leading DPI products utilizing traditional dry powder formulation technologies. Asmanex^®^ Twisthaler^®^ contains spheronized particles of mometasone furoate, while Flovent^®^ Diskus^®^ contains a blend of fluticasone propionate with coarse lactose carrier particles [38,76]. Both formulations exhibit a bimodal APSD with most of the dose (~80%) deposited in the non-respirable “large particle fraction”. Deposition in this stage grouping is about 40-fold higher than is observed for CPI. Deposition in the “airways fraction” on stages 3 to 5 is about 4 to 5-fold lower than CPI. Deposition on stages 4 and 5, a metric for small airways delivery, was about 7 to 8-fold lower than CPI. In essence, the coarse mode of the Asmanex and Flovent products has been shifted to particles of a respirable size in CPI. Equivalent fine particle deposition in the airways fraction would result in ~200-fold decreases in deposition in the large particle fraction.

The bottom row of products in Figure 9 is made up of formulations comprising extrafine drug particles with MMAD values of ~1 μm. These include two solution pMDIs (QVAR^®^ and Alvesco^®^) and a dry powder formulation (Foster^®^ Nexthaler^®^) [70,71,72,76]. QVAR and Foster Nexthaler deliver beclomethasone dipropionate, while Alvesco delivers ciclesonide. These three formulations also exhibit bimodal APSDs with between 24% and 35% deposition in the “large particle fraction” (coarse mode). Although not pictured, these formulations also exhibit higher deposition in the device (e.g., ~29% for QVAR, ~20% for Alvesco, 12–17% for Foster Nexthaler, versus only 6% for CPI) [70,71,72,76]. A large percentage of their dose (32–54%) deposits in the “very fine particle fraction” on stage 6 to MOC versus 19.6% for CPI. These formulations deposit a modest amount of their dose (19–33%) on stages 3 and 5 versus 78% for CPI. Deposition in the small airways fraction was 19–27% vs. 75% for CPI. Hence, the dry powder CPI formulation may improve targeting to the large and small airways relative to both fine and extrafine ICS formulations, while potentially reducing unwanted extrathoracic and alveolar deposition. The improved targeting to the airways should enable significant reductions in nominal dose (i.e., steroid sparing), a feature of importance to all patients, especially pediatric subjects.

### 4.4. Minimum Flow Rate

DPIs are often classified in terms of the minimum flow rate that achieves acceptable powder dispersion and efficacy in vivo [77,78,79].

Dispersion of the drug from the carrier or spheronized particle agglomerate depends critically on the pressure drop patients achieve through their dry powder inhaler during inhalation. Pediatric, geriatric, and female patients have reduced muscle strength, and sometimes may be unable to generate the inspiratory pressures needed to achieve effective drug dispersion [77,79]. Mahler suggests that a flow rate of 60 L min^−1^ is required for effective delivery with a DPI in COPD patients [79]. This rule-of-thumb neglects the impact of variations in device resistance on the flow rates achievable in various DPIs. For this reason, it has been proposed that pressure drop is a better metric than flow rate for comparing DPIs [55]. It was further proposed that if patients can achieve a pressure drop of ~1 kPa, that they can effectively use a dry powder inhaler [55]. For the drug-device combinations detailed herein, flow rates as low as ~20 L min^−1^ (1 kPa) were demonstrated to effectively fluidize and disperse CPI. Indeed spray-dried porous particles with high delivery efficiency have demonstrated lung delivery that is independent of pressure drop down to pressure drops as low as 0.2 kPa [8,49]. The “flow rate” limitations described in the literature for DPIs are more of a reflection of the limitations of LB and SPH formulations rather than an inherent limitation of DPIs.

## 5. Conclusions

- Adhesive mixtures comprising extrafine ciclesonide crystals and crystalline nanoleucine carrier particles achieve TLD values that are 93% and 87% of the ED in adult and child anatomical throat models, respectively.
- The TLD measured in the throat models was independent of flow rate and relative humidity.
- In an NGI, deposition of CPI in the “large particle fraction” comprising the induction port, stage 1 and stage 2 was just 2.3% of the emitted dose. In the “airways fraction” comprising stages 3 through stage 5, deposition was 78.1%. In the “very fine fraction” comprising stages 6 to MOC, deposition was 19.6%.
- Lung targeting, expressed as the ratio of the TLD/URT deposition was significantly greater than current marketed ICS therapeutics.
- The nanoleucine carrier technology represents an alternative to traditional lactose blends and solution pMDIs, with improved ability to target drug to the large and small airways in the lungs, while minimizing extrathoracic and alveolar delivery.

## 6. Patents

The material presented in this manuscript is the subject of a pending PCT patent application PCT/US2020/036944 submitted 10 Jun 2020. The application was published as: Miller, D.P., Tarara, T.E., Weers, J.G. Carrier-based formulations and related methods. WO2020251983A1 on 17 Dec 2020.

## Figures and Tables

**Table 1:** Properties of ciclesonide powder for inhalation.

Metric	Method	Mean ± SD
Ciclesonide assay (μg/mg)	RP-HPLC	9.6 ± 0.1
Geometric size $X_{10}$ (μm)	Laser diffraction	0.79 ± 0.01
$X_{50}$ (μm)	Laser diffraction	1.70 ± 0.02
$X_{90}$ (μm)	Laser diffraction	3.16 ± 0.05
Tapped density (g/cm^3^)	Tapped density	0.047 ± 0.001
$D_{a}$ (nm)	Calculated	370
Water content (% *w*/*w*)	DVS	< 0.3 (90% RH)
ICS physical form	XRPD	Crystalline
Leucine physical form	XRPD	Crystalline

**Table 2:** Aerosol performance metrics for CPI.

Metric	Method	Mean ± SD
Emitted dose (% ND) ^a^	DUSA / RP-HPLC	94.0 ± 0.7
Fine particle fraction < 5 μm (% ED) ^b^	NGI / RP-HPLC	96.8
Fine particle fraction S4-F (% ED) ^b^	NGI / RP-HPLC	94.5
Mass median aerodynamic diameter (μm) ^b^	NGI / RP-HPLC	1.66
Geometric standard deviation ^b^	NGI / RP-HPLC	1.57
Mass median impaction parameter (μm^2^ Lmin^-1^) ^b^	NGI / RP-HPLC	115.8
Total lung dose (% ED) ^a^	Alberta idealized throat AIT / RP-HPLC	93.0
Total lung dose (% ED) ^a^	Idealized child throat ICT / RP-HPLC	86.5
Q index (%) ^a^	ICT / RP-HPLC	−3.9
TLD 75% RH / TLD 40% RH ^b^	ICT / RP-HPLC	0.99

^a^ ΔP = 2 kPa, V = 4 L (AOS DPI); ^b^ ΔP = 4 kPa, V = 4 L (AOS DPI).

**Table 3:** Comparison of the stage distributions of various ICS formulations in an NGI [38,70,71,76].

Aerosol Metric	Mean $d_{a}^{2}Q\;\mathrm{Cutoff}\;(\mu m^{2}\;L\;\min^{-1})$	Asmanex Twisthaler (%ED)	Flovent Diskus (%ED)	Alvesco pMDI (%ED)	QVAR pMDI (%ED)	Foster Nexthaler (%ED)	CPI AOS (%ED)
Stage Grouping (T-S2)	> 1176	81.7	79.6	28.6	24.0	35.0	2.3
Stage Grouping (S3-S5)	56–1176	16.6	18.3	19.2	22.5	33.3	78.1
Stage Grouping (S4-S5)	56–467	10.9	9.0	19.2	21.5	26.5	74.9
Stage Grouping (S6-F)	< 56	2.7	1.2	52.2	53.5	31.8	19.6
Stage Grouping (S7-F)	< 21	1.7	0.1	26.7	29.5	18.6	4.4
MMAD (μm)	/	2.6	2.8	1.0	0.6	1.2	1.7
MMIP (μm^2^ L min^−1^)	/	394.8	704.2	29.0	30.9	61.8	115.8
Drug APSD	/	Fine	Fine	Extrafine	Extrafine	Extrafine	Extrafine

## Data Availability

Not applicable.

## Abbreviations

The following abbreviations are used in this manuscript.

AOS^®^ DPI	Axial oscillating sphere dry powder inhaler
APSD	Aerodynamic particle size distribution
CIC	Ciclesonide
COPD	Chronic obstructive pulmonary disease
CPI	Ciclesonide powder for inhalation
CT	Computed tomography
D	Diameter of the airspace
Da	Median aerodynamic diameter for primary particles
$d_{a}$	Aerodynamic diameter
$d_{a}^{2}Q$	Impaction parameter
$d_{g}$	Geometric diameter
$d_{p}$	Particle diameter
dm/dt	Change in mass per unit time
DSPC	Distearoylphosphatidylcholine
DUSA	Dose unit sampling apparatus
DVS	Dynamic vapor sorption
ED	Emitted dose
FPF_<5 μm_	Fine particle fraction less than 5 μm
g	Acceleration due to gravity
ICS	Inhaled corticosteroid
ICT	Idealized child throat
IP	Induction port
LEU	Leucine
LB	Lactose blend
LRT	Lower respiratory tract
MIP	Maximum inspiratory pressure
MMAD	Mass median aerodynamic diameter
MMIP	Mass median impaction parameter
MOC	Micro orifice collector
ND	Nominal dose
NP	Nanoparticles
PFOB	Perfluorooctyl bromide, Perflubron USP
RH	Relative humidity
RP-HPLC	Reverse phase-High performance liquid chromatography
SPH	Spheronized particles
Stk	Stokes Number
TLD	Total lung dose
URT	Upper respiratory tract
USP	United States Pharmacopeia
UV	Ultraviolet
X_10_	Diameter for which 10% of the particles have a smaller size
X_50_	Diameter for which 50% of the particles have a smaller size
X_90_	Diameter for which 90% of the particles have a smaller size
XRPD	X-ray powder diffraction
$\rho_{p}$	Particle density
$\rho_{tapped}$	Tapped density
μ	Dynamic viscosity
u	Linear velocity
ν	Terminal settling velocity
